# An imperfect “PAST” Lessons learned from the National Review of Asthma Deaths (NRAD) UK

**DOI:** 10.1186/s12931-016-0393-9

**Published:** 2016-07-19

**Authors:** Shuaib Nasser

**Affiliations:** Department of Allergy and Respiratory Medicine, Cambridge University Hospitals NHS Foundation Trust, Cambridge, CB2 0QQ UK

## Abstract

Asthma deaths are a barometer of the quality of asthma care. The principal care for patients with severe asthma is often a joint partnership between primary and secondary services. Communication between the two services determines the effectiveness of treatment. Undertaking an audit on asthma in either primary or separately in secondary care is a relatively straightforward process. However, when the audit spans both primary and secondary care in a country as large as the United Kingdom which is further sub-divided into the separate healthcare systems of England, Wales, Scotland, and Northern Island, then the audit becomes considerably more challenging. The National Review of Asthma Deaths (NRAD) reported in May 2014 was a confidential enquiry tasked with identifying circumstances surrounding asthma deaths across the whole of the UK, in order to ascertain avoidable factors and make recommendations to improve care and reduce future asthma deaths (Why asthma still kills: the National Review of Asthma Deaths (NRAD) Confidential Enquiry report, 2014, http://www.rcplondon.ac.uk/sites/default/files/why-asthma-still-kills-full-report.pdf). The idea for NRAD arose from a longstanding East of England confidential enquiry started in 1988 by Dr Brian Harrison and then handed onto me in 2001 until funding for the national review of asthma deaths was secured in 2010.

In the UK one in eight or 5.4 million of the population receives treatment for asthma with 65,000 hospital admissions each year representing one of the highest prevalence rates for asthma in the world. The number of deaths has plateaued at approximately 1,200 deaths per year with a gradual trend for reduced asthma mortality in recent years. The East of England Confidential Enquiry on which NRAD [1] was modelled had highlighted that the majority of asthma deaths were preventable with triggers in the young distinct to those found in an older population [2, 3]. Previous confidential enquiries had also reported that many asthma deaths occurred in patients who did not have a history of severe asthma. Earlier audits had commented on preventable factors but these had focussed mainly to psychosocial factors contributing to poor compliance and sub-optimal attendance at hospital and primary care appointments [4]. Other audits had highlighted that the fatal asthma attack was often of sudden onset [5, 6] which may have been responsible for the delay in calling for help. A significant proportion of patients had been hospitalised in the year before death suggesting on-going poor control but also highlighting opportunities for intervention and prevention of death [6, 7]. There was concern about underuse of objective recordings and delay in the use of mechanical ventilation [7]. One audit reporting on asthma deaths had usefully identified the relationship between peak flow variability and risk of death [8]. Some studies highlighted the issue of overuse of bronchodilator therapies in patients with poorly controlled asthma who subsequently died [9]. Many of these reports recommended better treatment of the final attack before death and suggested methods for improving compliance with therapy. The East of England Confidential Enquiry however did not view each asthma death as a single missed opportunity, rather as a series of failed opportunities principally because of a lack of individual asthma phenotyping. The lack of understanding of an individual’s underlying asthma mechanism results in triggers remaining unidentified and patients are therefore unable to anticipate and prevent exacerbations. Ineffective management can lead to mistrust of healthcare, a breakdown of communication and ultimately hampers compliance.

Asthma deaths occurring in the 12 months between February 2012 and January 2013 were identified from government statistic agencies. Asthma deaths recorded on the death certificate and coded as the underlying cause of death were included. More stringent criteria were used in those patients aged over 75 due to co-morbidities contributing to death. Using these criteria 900 deaths were identified. Further screening excluded those that did not have asthma or asthma was very unlikely to have contributed to death. In some cases insufficient information was available for a detailed review or clinicians refused to provide information. Eventually 195 cases were identified as having died from asthma and a detailed examination of their medical history was undertaken from primary and secondary care records. The review panel comprised 174 volunteer clinical assessors made up of paediatricians, adult physicians, general practitioners, pharmacists, and nurses with an interest in respiratory medicine.

## Key findings

The median age at the time of initial diagnosis of asthma was 37 years in the 195 deaths, and 69 % were diagnosed with asthma after the age of 15 years (Table 1). A number of avoidable factors were related to patients themselves, or their environment including non-adherence to medical advice and tobacco smoking or exposure to second hand smoke in the home. The expert clinical assessors identified that 46 % of the deaths could be prevented with alternative management if clinicians had better knowledge and implemented asthma guidelines. Psychosocial factors have been seen as an important contributor of asthma deaths and 16 % of asthma deaths had mental health issues for example anxiety and depression, social isolation was seen in 7 % and problems with substance abuse recorded in 6 %.

**Table 1:** Characteristics of NRAD patients who died from asthma

Duration of asthma (*n* = 104)	Median 11y (0-62y)
Age at diagnosis (*n* = 102)	Median 37y (10 months – 90y)
Age at death (*n* = 193)	Median 58 yrs (range 4 – 97 yrs)
Severity of asthma (*n* = 155)	Mild 9 %-
	Moderate- 49 %
	Severe- 39 %
Previous asthma hospital admissions	Hospital ever 47 %
	Intensive care ever 15 %
	Ventilation ever 7 %
Asthma triggers	Recorded in 49 %
History of Allergy	Recorded in 41 %
Evidence of eosinophilia	Recorded in 15 %

### Previous asthma severity

**Fig. 1:**
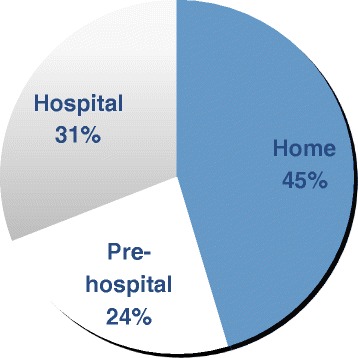
Location of death

Forty five per cent died without seeking medical help and only 31 % made it to hospital before death (Fig. 1). Only 43 % of the people who died from asthma had been under specialist supervision in the 12 months prior to death. Fewer than half had a history of previous hospital admission for asthma, although 10 % died within 28 days of discharge from hospital after treatment for asthma. 21 % had attended a hospital Emergency Department (ED) in the year before death and 10 % on more than one occasion. Of the 125 patients for whom severity could be estimated, only 39 % appeared to have severe asthma and 9 % were being treated for mild asthma. These statistics highlight that approximately half of the people who die from asthma are known to specialist services because of previous hospital admissions, ED visits, or because they have a history of severe asthma requiring specialist input. More surprising however are the 50 % of people who die from asthma who remain undetected either because they have a habit of avoiding medical help during exacerbations, or they are poor at recognising their own asthma symptoms, or they die after their first asthma exacerbation. It is this group that require the closest scrutiny, but often medical records are sparse, lending few clues.

### Taking treatment correctly

Personalised asthma action plans (PAAPs) are acknowledged to improve asthma care but were provided for only 23 % confirming that PAAPs had not even been provided for each of the 39 % considered to have severe asthma. Approximately 40 % had been prescribed more than 12 short acting reliever inhalers in the year before death, and a similar percentage had been issued with fewer than 4 inhaled corticosteroid inhalers in the 12 months before death. The proportion of overlap between these two groups of patients was not known. 14 % of those who died had been prescribed a single component long acting beta agonist (LABA) at the time of death and at least 3 % were taking a LABA without inhaled corticosteroid. This single statistic lends considerable concern to the practice of prescribing single device LABAs and inhaled corticosteroids separately, as it allows the possibility of prescription errors or confusion with treatment.

## Recommendations

NRAD concluded that a significant number of asthma deaths can be averted with implementation of the following small number of key recommendations.

### Asthma phenotype

The common misconception that an individual’s asthma phenotype or triggers are unimportant continues to be propagated in clinical practice despite international guidelines [10]. Exacerbating factors or triggers were documented in only half the patients. There was a possible history of atopy in 50 % and at least 15 % had evidence of eosinophilia on blood count. Until phenotyping becomes routine it is unlikely that asthma care will improve or deaths reduced significantly. All patients diagnosed with asthma should undergo clinical phenotyping in order to identify likely triggers, and these include pollens and fungal spores in those with predominantly sudden-onset attacks or seasonal symptoms especially in atopic subjects under the age of 45y [11, 12]. In older subjects winter exacerbations starting after a respiratory tract infection or sinusitis are more common. Patients with predictable seasonal exacerbations should undergo their annual asthma review a month before their worst season (Table 2).

**Table 2:** Recommendations to reduce risk of asthma death

Risk Factor	Recommendation
1. 45 % died at home without calling for help and Personal Asthma Action Plans (PAAPs) had only been issued to 23 %	All patients with asthma to be issued with PAAPs to include
	• asthma triggers,
	• current therapy,
	• advice on treatment escalation and
	• how to call for help
2. Failure to phenotype asthma or identify triggers in >51 %	Asthma clinical phenotyping to identify likely triggers and underlying mechanism of asthma
3. No evidence of a review in general practice in the last yr for 43 %	Structured annual review of asthma control preferably timed a month before expected seasonal exacerbation
4. 10 % died within 28d of hospital discharge and 21 % died within 12mo of attendance in ED	Follow-up arranged following every attendance in ED and secondary care follow-up after each hospital admission for asthma
5. Excessive prescribing of SABA (39 % issued with >12 devices in previous 12mo) inadequate uptake of ICS with 38 % issued <4 devices in previous 12 mo	Electronic surveillance of inhaler prescribing in primary care to identify those prescribed >12 short-acting beta-agonist or < 4 corticosteroid inhaler devices in previous yr

### Self-management

Each patient with asthma should have a written and personalised asthma action plan recording their likely phenotype (e.g. allergic, non-allergic, eosinophilic, infective) and clinical triggers recorded. The plan should include details of current treatment and how to manage exacerbations. The fact that only 50 % of those who died from asthma called for help underscores the importance of clear guidance on how to call for help within the asthma action plan. Involvement of patients in their own management should be encouraged and this is easier once individual phenotypes have been identified and patients informed of their identified triggers. Examples of ways to encourage self-management include increasing medication before the start of the hay fever season, avoiding non-steroidal anti-inflammatory drugs (NSAIDs) in susceptible individuals, or the early use of oral corticosteroids with viral- or allergen-induced exacerbations.

### Follow-up and electronic surveillance

Patients frequently attending the Emergency Department or those who have been recently discharged from hospital should have timely follow up, and the former group may require a degree of persuasion to attend appointments. Every patient should have a structured annual review of asthma control and treatment with emphasis on preventing future exacerbations. Where loss of control is identified, immediate escalation of treatment is required and arrangements made for follow up. An innovative NRAD recommendation was electronic surveillance of inhaler prescribing in primary care in order to identify those patients prescribed more than 12 short-acting beta-agonist (SABA) or fewer than 4 corticosteroid inhaler devices in the previous year. This allows the possibility of preventative action in those demonstrating a lack of understanding or poor compliance. The prescription of long acting beta-agonists in a single device should no longer be acceptable in asthma.

### Remember “PAST”

The Eastern Region Confidential Enquiry into Asthma Deaths coined the acronym “PAST” to reflect the key measures required to reduce asthma deaths.

P = personalised asthma action plans

A = asthma phenotype

S = structured asthma review

T = therapy - avoiding:

- Excess SABAs,
- Non-combination LABAs, and
- Inadequate inhaled corticosteroids.

### The future

An important question that NRAD failed to answer was why do people die if they are considered to have mild to moderate asthma? The study did not identify the individual characteristics, phenotype, and behaviour patterns of this group and therefore further study will be necessary to understand whether this group are poorly compliant, have specific triggers leading to sudden death, or if they simply die following their first severe exacerbation. Preventing future deaths will require a better understanding of this group in particular (Table 3).

**Table 3:** Risk factors for asthma death identified from NRAD

Risk Factor
Lack of appropriate action by health care professional or patient during asthma exacerbation
Excess prescriptions of reliever medication (>12 Short acting beta-agonist bronchodilator devices in previous 12 months)
Poor adherence with inhaled corticosteroids (<4 in previous 12 months)
Use of long acting beta-agonist bronchodilators without Inhaled corticosteroids
Hospital admission for asthma in the month before death
Two or more attendances at A&E (ED) in year before death
Failure to phenotype asthma or identify triggers for asthma attacks
Asthma diagnosed in adulthood

## Abbreviations

ED: emergency department
LABA: long acting beta agonist
NRAD: national review of asthma deaths
NSAID: non-steroidal anti-inflammatory drugs
PAAP: personal...ised (personalised) asthma action plans
SABA: short-acting beta-agonist
